# Bio-organic fertilizers promote yield, chemical composition, and antioxidant and antimicrobial activities of essential oil in fennel (*Foeniculum vulgare*) seeds

**DOI:** 10.1038/s41598-023-40579-7

**Published:** 2023-08-25

**Authors:** Ahmed S. Abdelbaky, Abir M. H. A. Mohamed, Taia A. Abd El-Mageed, Mostafa M. Rady, Fatma Alshehri, Mohamed T. El-Saadony, Synan F. AbuQamar, Khaled A. El-Tarabily, Omar A. A. Al-Elwany

**Affiliations:** 1 Department of Biochemistry, Faculty of Agriculture, Fayoum University, Fayoum, 63514 Egypt; 2 Department of Agricultural Microbiology, Faculty of Agriculture, Fayoum University, Fayoum, 63514 Egypt; 3 Department of Soil and Water, Faculty of Agriculture, Fayoum University, Fayoum, 63514 Egypt; 4 Department of Botany, Faculty of Agriculture, Fayoum University, Fayoum, 63514 Egypt; 5 Department of Biology, College of Sciences, Princess Nourah bint Abdulrahman University, Riyadh, 11671 Saudi Arabia; 6 Department of Agricultural Microbiology, Faculty of Agriculture, Zagazig University, Zagazig, 44511 Egypt; 7 Department of Biology, College of Science, United Arab Emirates University, Al Ain, 15551 United Arab Emirates; 8 Department of Horticulture, Faculty of Agriculture, Fayoum University, Fayoum, 63514 Egypt

**Keywords:** Plant sciences, Plant physiology

## Abstract

The aromatic fennel plant (*Foeniculum vulgare* Miller) is cultivated worldwide due to its high nutritional and medicinal values. The aim of the current study was to determine the effect of the application of bio-organic fertilization (BOF), farmyard manure (FM) or poultry manure (PM), either individually or combined with *Lactobacillus plantarum* (LP) and/or *Lactococcus lactis* (LL) on the yield, chemical composition, and antioxidative and antimicrobial activities of fennel seed essential oil (FSEO). In general, PM + LP + LL and FM + LP + LL showed the best results compared to any of the applications of BOF. Among the seventeen identified FSEO components, *trans*-anethole (78.90 and 91.4%), fenchone (3.35 and 10.10%), limonene (2.94 and 8.62%), and estragole (0.50 and 4.29%) were highly abundant in PM + LP + LL and FM + LP + LL, respectively. In addition, PM + LP + LL and FM + LP + LL exhibited the lowest half-maximal inhibitory concentration (IC_50_) values of 8.11 and 9.01 μg mL^−1^, respectively, compared to l-ascorbic acid (IC_50_ = 35.90 μg mL^−1^). We also observed a significant (*P* > 0.05) difference in the free radical scavenging activity of FSEO in the triple treatments. The in vitro study using FSEO obtained from PM + LP + LL or FM + LP + LL showed the largest inhibition zones against all tested Gram positive and Gram negative bacterial strains as well as pathogenic fungi. This suggests that the triple application has suppressive effects against a wide range of foodborne bacterial and fungal pathogens. This study provides the first in-depth analysis of Egyptian fennel seeds processed utilizing BOF treatments, yielding high-quality FSEO that could be used in industrial applications.

## Introduction

Essential oils (EOs) extracted from medicinal and aromatic plants (MAPs) have been widely used for their antispasmodic, sedative, digestive, cardiotonic, diuretic, and tonic effects in alternative medicine^1^. They are routinely added to foods and are usually acknowledged safe when these plants and/or their EOs are farmed organically using certified procedures. In addition, EOs have long been used as flavorings in food industry, as well as many other applications in cosmetics, hygiene products, pharmaceutical medications, and fragrances^2–4^. The natural antioxidant effects of EOs can also be used as alternative food preservatives^5,6^.

In 2021, 2.3 million hectares of MAPs were harvested worldwide, yielding over 2.7 metric tons of seeds^7^. Egypt has contributed to more than 32,000 hectares of the harvested area—the vast majority of which is distributed across areas negatively impacted by salinity—yielding about 29,000 tons of fruitful seeds. On average, Egypt's exports of MAPs bring in around $160 million per year^8^, while the trade value of Egyptian exported EOs increased from $33 million in 2016 to $60 million in 2020^9^.

Fennel (*Foeniculum vulgare* Miller) is one of the MAPs that is commonly used as a kitchen herb worldwide^10^. This member of the Apiaceae family is widely grown in arid and semi-arid areas, including Egypt, particularly in the Fayoum governorate^11,12^. Compounds with antibacterial, hepatic, and antioxidant properties have been identified in the edible parts of fennel^13^. Stems and leaves of fennel plants are rich in vitamins A, B and C, as well as potassium (K) and calcium, all of which play an important role in different metabolic processes^10^.

Fennel seeds essential oil (FSEO) is a popular flavoring agent used in a wide variety of foods (e.g., bread, cheese, pickles, pastries, and beverages), cosmetics and pharmaceutical products^14,15^. In addition, several studies have reported that FSEO show biological activites, such as hepatoprotective^16^, antioxidant^17^, antibacterial^2^, antifungal^18^, anti-diabetic^19^, anti-neurological^20^ and anticancer^21^. It has also been reported that trans-anethole, estragole, fenchone and limonene are the primary components of FSEO^5,22,23^.

Saline calcareous soils with electrical conductivity of saturated soil extract (ECe) greater than 4 dS m^−1^^24^, high content of CaCO_3_ and/or MgCO_3_ (14–17%), low organic matter, and alkaline pH are commonly found in many dry and semi-arid regions, including the Mediterranean^25^. This type of soil is often associated with reducing nutrient solubility and availability, particularly phosphorus (P) and crop yield^26^. In addition, high ECe-CaCO_3_ alters the physical properties of saline-calcareous soils, including the amount of water available to plants and the surface crust^27^.

Due to the increasing demand on their products, there is a growing interest in the cultivation of MAPs around the world, especially those which practice organic agriculture^28^. Organic fertilizers can provide all nutrients that are required for MAPs. Composting and recycling of organic waste, such as food scraps, plant matter and animal byproducts, are examples of organic fertilizers, which have positive impacts on soil structure, in addition for being safe to the environment and human health^28^.

Organic manures (OM) can improve the physical, chemical, and biological properties of soils. These organic amendments have beneficial influence on the structure, stability, pH, cationic exchange capacity, nutrient availability and microbial activity of soils^29,30^. Bio-fertilizers, including OM, can potentially be used to replace chemical fertilizers for sustainable agriculture systems^30^.

Although FSEO has been the subject in several studies, the phytochemical composition, antioxidative and antimicrobial properties of Egyptian fennel grown in saline-calcareous soil and treated with bio-organic fertilization (BOF) have not been investigated yet. This study was, therefore, carried out to examine the impact of BOF treatments on production FSEO of Egyptian fennel plants in saline-calcareous soil. The antioxidative and antimicrobial properties of the resultant FSEO against human foodborne pathogenic bacteria and fungi were in vitro determined.

## Materials and methods

### Experimental location

Two field-scale trials were performed in 2019/2020 and 2020/2021. A factorial layout with a randomized complete block design (RCBD) was applied. The experiments were carried out on a plot of soil at a research farm in Fayoum governorate (29° 17′N; 30° 53′E), Egypt. Mean temperature throughout the experimental period (from October to May) were 25 ± 3 °C/10 ± 2 °C for average day/night temperatures; average relative humidity of 75 ± 4%. Natural sunlight (11 h for average daylight length) was sufficient for all growth stages of fennel plnats.

### Land properties

According to the climatic spectrum and aridity index^31^, the experimental site was in arid area. Soil was classified as typic tropopsamments, siliceous, and hyperthermic based on Soil Survey Staff USDA^32^. Soil samples were collected from the upper soil layer (0.0–0.2 m in depth). All physio-chemical analysis of the studied soil was carried out according to the methods described by^33,34^. The soil used in this study was saline calcareous, sandy loam in texture (74.66% sand, 12.15% silt, and 13.19% clay). The ECe was 6.92 dS m^−1^, CaCO_3_ = 13.8%, pH = 7.64, OM = 0.89%, and the available N was 0.016% (Table S1).

### Plant materials

Seeds of *F. vulgare* were obtained from the Institute of Medicinal and Aromatic Plants, Agricultural Research Center (ARC), Giza, Egypt. Fennel seeds were hand-bedded in hills 0.3 m apart (3–5 seeds hill^−1^), on October 27th of both seasons. Twenty-one days after germination (DAG), hills were thinned to 2–3 seedlings, and re-thinned again at 45 DAG to maintain only the strongest plant hill^−1^. The experimental site was fertilized with the recommended doses of 75 kg P_2_O_5_ ha^−1^ as (P), two equal applications of 150 kg N ha^−1^ applied at 45 and 75 DAG, and 50 kg K_2_O ha^−1^ (K) totally applied with the second application of N-fertilization. Disper Complex GS (Chelated-Microelements, 0.5 g L^−1^) were sprayed on the flowage of fennel crop at 40 and 70 DAG, purchased from Sphinx International Trade Co., Nasr City, Egypt. All the matured fennel crop were hand-picked up on May 8 in this 2-year study. The use of plants/plant parts, in the present study, complies with the international, national and/or institutional guidelines.

### Treatments and experimental setup

Two organic fertilizers comprising of farmyard manure (FM) or poultry manure (PM) were purchased from cattle and poultry producers (private farms) based in Fayoum city, Fayoum governorate, Egypt.

The chemical properties of FM and PM are presented in Table (S2). FM and PM were applied individually at rates of 25 and 20 m^3^ ha^−1^, respectively, as commercial agronomic regional practices of fennel, or applied in combination with the two lactic acid bacteria (LAB) strains, *Lactobacillus plantarum* (LP) and *Lactococcus lactis* (LL). LP and LL were applied individually or in a mixture as seed inoculation.

In total, nine treatments were applied as the following: (1) C, control, no seed or soil treatment; (2) FM, soil treatment with farmyard manure; (3) FM + LP, soil treatment with farmyard manure + seed treatment with *L. plantarum*; (4) FM + LL, soil treatment with farmyard manure + seed treatment with *L. lactis*; (5) FM + LP + LL, soil treatment with farmyard manure + seed treatment with *L. plantarum* + *L. lactis*; (6) PM, soil treatment with poultry manure; (7) PM + LP, soil treatment with poultry manure + seed treatment with *L. plantarum*; (8) PM + LL, soil treatment with poultry manure + seed treatment with *L. lactis*; and (9) PM + LP + LL, soil treatment with poultry manure + seed treatment with *L. plantarum* + *L. lactis*.

Each treatment was applied three times, with a total of 27 plots. The area of the experimental plot was 3 m in length × 3 m row width (9 m^2^). Each plot contained 5 lines, each 3.0 m in length and 60 cm apart.

### Bacterial strains

Two bacterial strains (*L. plantarum* subsp*. plantarum* ATCC 14917 and *L. lactis* subsp*. lactis* ATCC 11454) obtained from the Department of Agricultural Microbiology and Biotechnology, Ain Shams University, Egypt were used in the current study. Both strains were cultivated on de Man, Rogosa and Sharpe (MRS) agar (Lab M Limited, Lancashire, UK) and stored at 4 °C. Cell suspensions of bacterial strains were obtained by inoculation of each strain in double-strength MRS broth and cultivated overnight at 37 °C. The final concentration of cells reached 5 × 10^9^ colony forming units (CFU) mL^−1^. To inoculate fennel seeds, 100 mL of cell suspensions of each *Lactobacillus* strain was transferred to the 250 mL flask and stored overnight at 37 °C. Fennel seeds were inoculated with a cell suspension of either LP or LL (1:1).

### Extraction and analysis of FSEO

#### Extraction of FSEO

**Figure 1:**
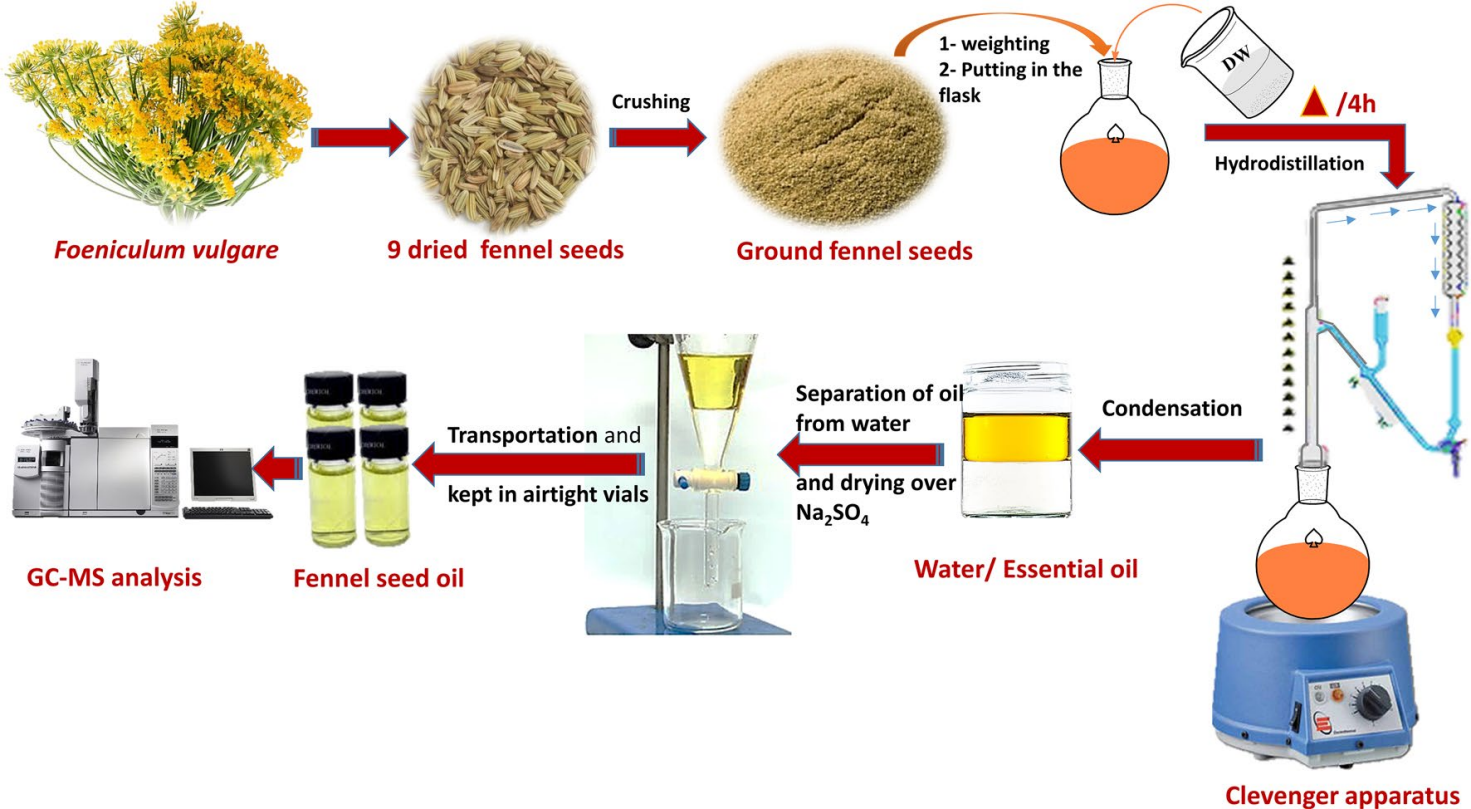
Flow chart of the hydro-distillation of fennel seed oil production process.

Air-dried fennel seeds powder (100 g) from each plot were subjected separately to hydrodistillation in 1 L of double distilled water (DDW) and boiled for 4 h in a Clevenger apparatus^35^. The extracted oils from each plot were dried over anhydrous sodium sulfate (Advent Chembio PVT. LTD, Mumbai, India) to eliminate any traces of moisture, then weighted and kept in air-tightly closed dark vials at − 80 °C until use (Fig. 1).

#### Analysis of FSEO

FSEO from each plot were analyzed by using trace gas chromatography (GC) (model GC1310-ISQ) mass spectrometry (MS; Thermo Scientific, Austin, TX, USA) equipped with TG-5MS column (30 m × 0.25 mm × 0.25 μm film thickness), with helium as a carrier gas at a constant flow rate of 1 mL min^−1^. The column oven temperature was initially held at 50 °C and then raised by 5 °C min^−1^ to 230 °C, held for 2 min, and raised to the final temperature of 290 °C at 30 °C min^−1^ and held for 2 min. The injector and MS, transfer line temperatures, were kept at 250 and 260 °C, respectively. The solvent delay was 3 min, and diluted samples of 1 µL were injected automatically using autosampler AS1300 coupled with GC in the split mode. In full scan mode, electron ionization mass spectra were collected at 70 eV ionization voltages over m/z 40–1000. The ion source temperature was set at 200 °C. The components were identified by comparison of their retention times and mass spectra with those of WILEY 09 and NIST 11 mass spectral databases.

### Determination of the antioxidant potential

The antioxidant effect of FSEO was evaluated by the assay of 2,2-diphenyl-1-picrylhydrazyl (DPPH;^36^) with slight modifications by^37^. Briefly, 2 mL of the stable free radical DPPH solution (Sigma–Aldrich Chemie GmbH, Taufkirchen, Germany) (0.003% dissolved in methanol) was added to 100 µL of each FSEO methanolic solution and l-ascorbic acid (as a positive control) with various concentrations (5, 10, 20, 40, 80, 160 and 320 μg mL^−1^).

After stirring vigorously for 1 min, the reaction mixture was kept at 35 ± 2 °C for 30 min in the dark. The decrease in absorbance was recorded at 517 nm via the U-2900 UV–Vis double-beam spectrophotometer (Hitachi, Tokyo, Japan). Three replications for each measurement were carried out. For each sample, the DPPH free radical scavenging activity (DPPH FRSA) was computed as:

$$\% {\text{ Inhibition }}\left( {{\text{DPPH}}\;{\text{FRSA}}} \right)\, = \,\left[ {\left( {{\text{Acs}} - {\text{Ats}}} \right)/{\text{Acs}}} \right]\, \times \,100.$$

where Acs, the absorbance of the control sample; Ats, the absorbance of the treatment sample. The half-maximal inhibitory concentration (IC_50_) values (the concentration required for 50% inhibition of viability) were assessed from the relationship FRSA curve versus concentrations of the curve of the respective sample.

### Determination of the antimicrobial effect

#### Microbial strains sources, culture conditions and inoculum preparation

The FSEO antimicrobial efficiency was tested against different bacterial and fungal strains, including two Gram positive bacteria, *Staphylococcus aureus* (ATCC 8095) and *Bacillus subtilis* (ATCC 13753), and two Gram negative bacteria*, Escherichia coli* (ATCC 25922) and *Pseudomonas aeruginosa* (ATCC10662). Two fungal strains (*Penicillium roqueforti* and *Aspergillus niger*) known for their food spoilage and mycotoxins production were also used in the present study.

All bacterial strains were obtained from the Agricultural Microbiology Department, Fayoum University, Egypt, while the Mycological Center, Assiut, Egypt provided the fungal strains. Bacterial cultures were cultured on the Luria–Bertani (LB) agar (Lab M), and the fungal cultures were cultivated on potato dextrose agar (PDA) (Lab M). All strains were stored at 4 °C and subcultured once a month.

Fungal cultures were grown on PDA for 7 days at 28 °C until good sporulation was obtained. For the preparation of fungal spore suspension, 5 mL of a sterile saline solution (0.85%) containing tween 80 (Sigma) (0.1%) was added to the surface of the cultures, followed by gentle scraping with a sterile needle. After settling down for 3 min, the homogeneous upper suspension was used as inocula. The tests were then carried out using a suspension containing 10^8^ spores mL^−1^. Bacterial inocula were prepared by inoculating the culture into a 50 mL LB broth medium (Lab M) in an Erlenmeyer flask. The flasks were incubated in a shaker incubator at 37 °C for 24 h at 150 rpm. Bacterial inoculum was adjusted to 10^7^ CFU mL^−1^; 0.5 Mac-Farland.

#### Disc-diffusion assay

Disc-diffusion assay^38^ was employed to determine the antimicrobial activity of FSEO against the tested strains. Solidified plates containing LB agar for bacterial strains and PDA for fungal strains were seeded with 0.2 mL from the inoculum suspension previously described. Different concentrations of FSEO were added to 9 mm Whatman #1 filter paper disks which were placed on the agar surface. The plates were left for 60 min to diffuse and then incubated at 37 °C for 24 h for bacteria and at 28 °C for 5 days for fungi. Antimicrobial activities were measured as the inhibition zone diameter around each disk. The antibiotics gentamycin and clotrimazole were used as a positive control for bacteria and fungi, respectively.

#### Effect of essential oils on hyphal morphology

The determination of the volatile FSEO effects on hyphal morphology was previously described^39^.

### Statistical analysis

All experiments were carried out with three replications for each FSEO concentration. Data were analyzed using the two-way analysis of variance (ANOVA) and Duncan’s multiple range test were used to determine the statistical significance at *P* < 0.05. For all statistical analyses, SPSS^®^ IPM^®^ statistical program (version 23, New York, USA) was used.

## Results and discussion

### FSEO yield

**Figure 2:**
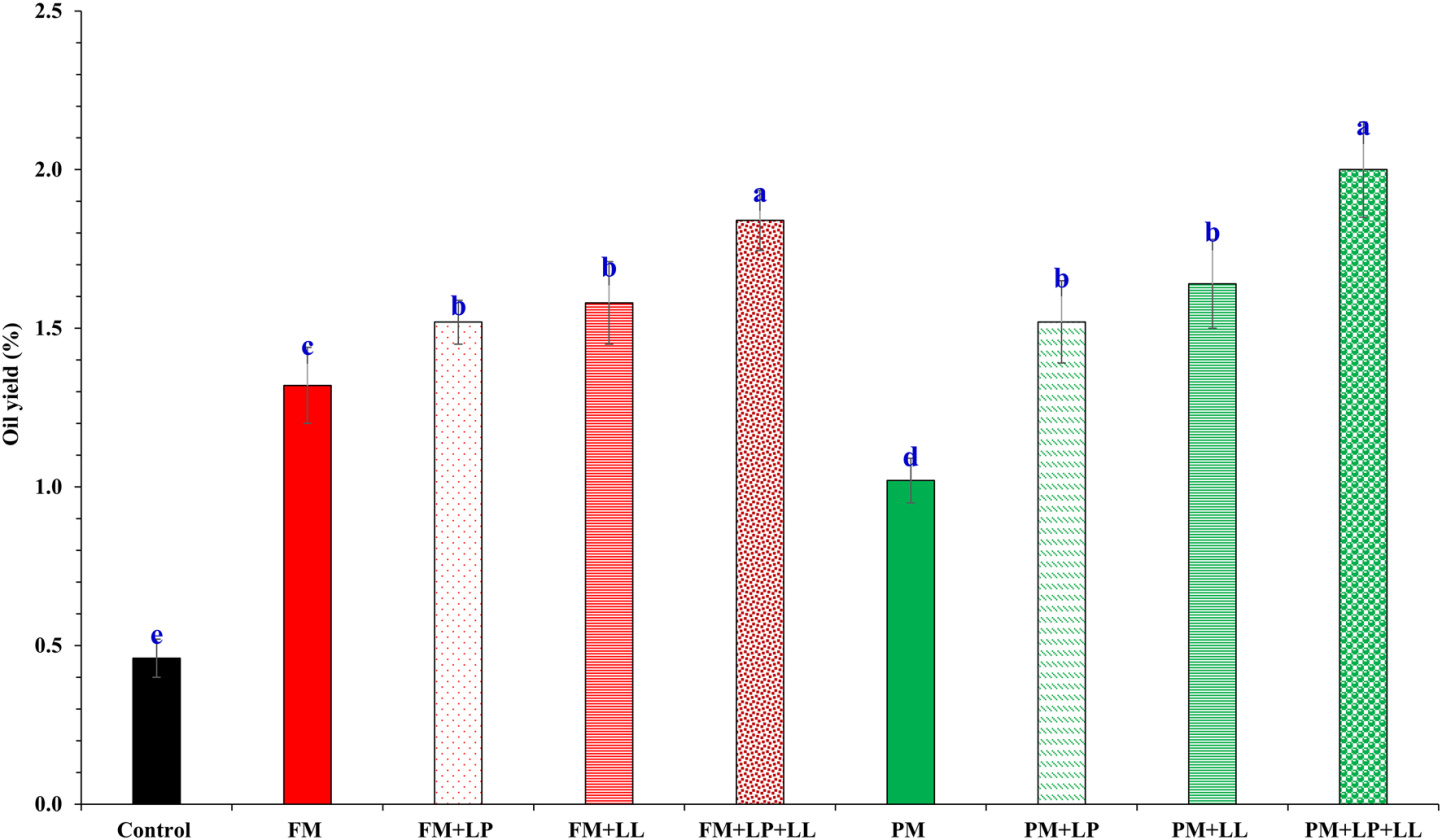
Effect of bio-organic fertilization on fennel (*Foeniculum vulgare*) seed oil yield (%) by hydro-distillation. Treatments were: (1) C, control, no seed or soil treatment; (2) FM, soil treatment with farmyard manure; (3) FM + LP, soil treatment with farmyard manure + seed treatment with *Lactobacillus plantarum*; (4) FM + LL, soil treatment with farmyard manure + seed treatment with *Lactococcus lactis*; (5) FM + LP + LL, soil treatment with farmyard manure + seed treatment with *Lactobacillus plantarum* + *Lactococcus lactis*; (6) PM, soil treatment with poultry manure; (7) PM + LP, soil treatment with poultry manure + seed treatment with *Lactobacillus plantarum*; (8) PM + LL, soil treatment with poultry manure + seed treatment with *Lactococcus lactis*; (9) PM + LP + LL, soil treatment with poultry manure + seed treatment with *Lactobacillus plantarum* + *Lactococcus lactis*. Bars represent standard error. Mean values followed by different letters are significantly (*P* < 0.05) different from each other according to Duncan’s multiple range test.

Among the treatments studied, FSEO yield was significantly different (*P* ≤ 0.05; Fig. 2) and the highest FSEO percentage (2.0 ± 0.15%) was obtained under combined application of PM + LP + LL, followed by FM + LP + LL (1.84 ± 0.10%), PM + LL (1.64 ± 0.14%) and FM + LL (1.58 ± 0.13%); while the lowest FSEO percentage was observed from the unfertilized control (0.46 ± 0.06%). It is worth mentioning that the PM + LP treatment maintained the same FSEO percentage (1.52 ± 0.13%) as FM + LP (1.52 ± 0.07%).

In Iran, FSEO content ranged from 2.7 to 4%^40^, but FSEO yield in Pakistan was 2.81%^41^. Although FSEO yield was 0.1% from Portugal^42^, the FSEO from 16 wild edible Tunisians *F. vulgare* ranged from 1.2 to 5.06%^43^. In addition, it was found the FSEO yield from Egyptian organic fennel was 1.6% without any treatments^44^. The content of EO can mainly be influenced by the environmental geographical conditions of the regions, climatic changes, the nature of the soil, and genetic factors^14^. Moreover, the technique and extraction process may have an effect^45^. According to^46^ effective agricultural and environmental practices would also help in enhancing the quality and yield of EOs.

### GC–MS analysis of FSEO

GC–MS analysis of FSEO led to the identification of 17 components, which represented 99.94–100% of the total composition belonging to hydrocarbons, alcohols, ethers, ketones, esters, amines, fatty acids, monoterpenes and sterols (Table 1; Fig. S1). Ethers represent the most available component in fennel seeds among all tested BOF treatments.

**Table 1:** Principal constituents of FSEO composition (%) obtained by GC–MS.

#	Component	RT (min)	MW	Treatment								
				1	2	3	4	5	6	7	8	9
				Area (%)								
Hydrocarbon												
1	Pentane, 3-methyl	5.06	86	0.53^c^	0.29^d^	0.71^a^	0.63^b^	0.53^c^	0.32^d^	0.56^c^	0.30^d^	0.31^d^
Alcohols												
2	Linalool	6.20	210	0.11^b^	0.07^cd^	0.05^de^	0.09^c^	0.03^e^	0.72^a^	0.04^e^	0.11^b^	0.07^cd^
3	(6-Hydroxymethyl-2,3-dimethylphenyl) methanol	37.00	166	0.03^bc^	0.02^c^	0.03^bc^	0.04^bc^	0.01^c^	0.11^a^	0.06^b^	0.03^bc^	0.03^bc^
Ethers												
4	(E)-anethole	10.65	148	81.3^g^	88.3^c^	84.2^e^	79.3^h^	89.2^b^	84.0^f^	84.8^d^	78.9^i^	91.4^a^
5	Estragole	12.50	148	0.50^f^	0.60^e^	0.99^c^	0.52^f^	0.54^f^	4.15^b^	0.71^d^	4.29^a^	0.52^f^
Ketones												
6	Fenchone	6.95	152	9.53^a^	3.35^g^	5.89^b^	9.55^a^	3.82^f^	5.55^d^	4.02^e^	5.82^c^	2.84^h^
Esters												
7	2-Myristynoyl pantetheine	21.88	484	0.01^c^	0.01^c^	0.01^c^	0.02^c^	0.06^b^	0.26^a^	0.02^c^	0.05^b^	0.03^c^
8	Hexadecanoic acid, methyl ester	27.89	270	0.37^d^	0.35^e^	0.49^b^	0.44^c^	0.38^d^	0.52^a^	0.44^c^	0.32^f^	0.34^e^
9	Cis-9-Octadecenoic acid, (2-phenyl-1,3-dioxolan-4-yl) methyl ester,	39.04	444	0.02^b^	0.01^c^	0.03^a^	0.03^a^	0.03^a^	0.03^a^	0.03^a^	0.02^b^	0.03^a^
10	9,12,15-Octadecatrienoic acid, 2-[(trimethylsilyl)oxy]-1-[[(trimethylsilyl)oxy]methyl]ethyl ester, (Z,Z,Z)	40.95	496	0.02^d^	0.03^c^	0.15^a^	0.01^e^	0.02^d^	0.15^a^	0.03^c^	0.02^d^	0.05^b^
Amines												
11	Dimethyldiphenyltethylidylpyrrolidine	18.17	277	0.06^c^	0.01^d^	0.01^d^	0.01^d^	0.02^d^	0.26^a^	0.03^d^	0.19^b^	0.07^c^
Fatty acids												
12	9-Octadecenoic acid (Z)-	31.06	282	0.81^c^	0.69^e^	1.02^b^	0.79^d^	0.97^b^	0.51^f^	1.22^a^	0.65^e^	0.46^g^
13	Cis-Oxiraneoctanoic acid, 3-octyl-,	33.56	298	0.04^bc^	0.01^d^	0.11^a^	0.09^a^	0..06^b^	0.02^d^	0.03^cd^	0.06^b^	0.01^d^
Monoterpene hydeocarbons												
14	Limonene	5.49	136	5.61^d^	5.91^c^	5.76^cd^	7.52^b^	3.87^e^	2.94^g^	7.38^b^	8.62^a^	3.51^f^
15	3-Pinanylamine	8.26	153	0.90^f^	0.24^e^	0.36^d^	0.75^a^	0.35^d^	0.35^d^	0.5^c^	0.55^b^	0.21^e^
Sterols												
16	Cholesta-8,24-dien-3-ol, 4-methyl-, (3á,4à)-	44.19	398	0.08^cde^	0.05^e^	0.15^a^	0.11^b^	0.10^bc^	0.02^f^	0.09^bcd^	0.06^e^	0.07^de^
17	Stigmast-5-en-3-ol, (3á,24s)-	47.12	414	0.01^c^	0.01^c^	0.01^c^	0.05^ab^	0.01^c^	0.06^a^	0.01^c^	0.01^c^	0.04^b^
				99.96	99.95	99.97	99.95	99.94	99.97	99.97	100	99.99

Treatments were: (1) C, control, no seed or soil treatment; (2) FM, soil treatment with farmyard manure; (3) FM + LP, soil treatment with farmyard manure + seed treatment with *Lactobacillus plantarum*; (4) FM + LL, soil treatment with farmyard manure + seed treatment with *Lactococcus lactis*; (5) FM + LP + LL, soil treatment with farmyard manure + seed treatment with *Lactobacillus plantarum* + *Lactococcus lactis*; (6) PM, soil treatment with poultry manure; (7) PM + LP, soil treatment with poultry manure + seed treatment with *Lactobacillus plantarum*; (8) PM + LL, soil treatment with poultry manure + seed treatment with *Lactococcus lactis*; (9) PM + LP + LL, soil treatment with poultry manure + seed treatment with *Lactobacillus plantarum* + *Lactococcus lactis.*

For each constitutes, means (n = 3) in the same horizontal row followed by different small letters differ significantly at *P* ≤ 0.05 according to analyzed by Duncan’s multiple range test.

FSEO, fennel seeds essential oil; GC–MS, gas chromatography–mass spectrometry; RT, retention time; MW, molecular weight.

#### Ethers

Ethers were the most prevalent class in all treatments applied, accounting for 79.82–91.92%, emphasizing their antioxidative and antibacterial properties, making them noteworthy dietary components^47^. Ethers reaching 91.92% and 89.74% of the total volatiles in the triple combination of PM + LP + LL and FM + LP + LL, respectively, compared to untreated control (81.8%). *(E)*-anethole and its isomer estragole (i.e., phenylpropanoid derivatives) are extensively found in different plants. In star anise (*Illicium anisatum *L.) and anise (*Pimpinella anisum *L.), *(E)*-anethole is the main volatile compound, while estragole is prevalent in sweet basil (*Ocimum basilicum* L.) and tarragon (*Artemisia dracunculus* L.)^48^. In the current study, (*E)*-anethole (No. 4, Table 1) was the major volatile compound in fennel seeds produced under all BOF treatments. The sweet, distinct, anise-like flavor that distinguishes fennel fruits could be contributed to *(E)*-anethole, which is also used as a flavoring and fragrance ingredient in the food industry and cosmetics^49^. In addition, *(E)*-anethole possesses various pharmacological properties, including anti-inflammatory, immunomodulatory, neuroprotective, and diabetic.

On contrast, estragole has no discernible effect on the total fennel aroma, albeit its high affinity to alkenylbenzenes (e.g., methyleugenol and safrole; Fig. S2) which are classified as carcinogens (Class 2B) according to the International Agency for Research on Cancer (IARC), which prompted the European Union (EU) to restrict utilizing estragole in nonalcoholic beverages to 10 mg kg^−1^^50^.

Recently, estragole has received attention due to its genotoxicity and hepatocarcinogenic properties^51^. These effects result from the 1′-hydroxyestragole sulfuric ester, an estragole metabolite, forming an adduct with DNA. Accordingly, the toxicity is not initiated by the parent compounds but by their highly reactive metabolites. On the other hand, it has been demonstrated that other plant components, such as flavonoid nevadensin can prevent the formation of estragole DNA adducts caused by sulfotransferase (SULT) that converts 1′-hydroxyestragole to the critical carcinogen 1′-sulfooxyestragole^52–54^.

In addition, the toxicokinetic of alkenylbenzenes, such as estragole versus trans-anethole, are influenced by structural differences in these compounds (Fig. S2). This influences the toxic (particularly genotoxic) potential of various alkenylbenzenes, which must be considered when evaluating the possible dangers associated with exposure to these chemicals^54^. Recognizing these threats, the European Medicines Agency has advised pregnant women, nursing mothers and young children to minimize the estragole supplementation. Eventhough no suzerainty has banned using the estragole-containing herbs, the European Union Commission has banned their use as food additives^55^.

#### Ketones

After ethers, the ketone fenchone (No. 6, Table 1) was the second major class of volatiles in all fennel treatments amounting 2.84–9.55%. In all treatments, only fenchone was found and present at a much higher level in FM + LL (9.55%) compared to that in FM + LP and PM + LL at 5.89 and 5.82%, respectively. It was, however, found at much lower levels in PM + LP + LL at 2.84%, probably due to the impact of high levels of (*E*)-anethole.

Our result agreed with a recently published report^56^ where ketones scored 7.52% when fennel plants were treated with humic acid. Due to fennel's bitter aftertaste, fenchone is utilized as a flavor for food owing to its camphor-like aroma^57^, in addition to its antifungal, acaricidal, and wound-healing properties^58^.

#### Monoterpene hydrocarbons (MTHCs)

After ethers and ketones, MTHCs were the plentiful third class in combinations (PM + LL, FM + LL and PM + LP) at 9.17, 8.27 and 7.88%, respectively, and to a lesser extent in control, FM and FM + LP (6.51–6.12%), and reached to almost 4.25% in the remaining treatments (Table 1). This is consistent with a report in which MTHCs were found to be 7.15% of the total^59^.

The major MTHCs identified was limonene (No. 14, Table 1) which was found at the highest level in PM + LL (8.62%) and FM + LL (7.52%), respectively. Limonene, a key component of citrus fruits, is an additive to numerous food products for its lemon-like flavor and anti-inflammatory properties against multiple intestinal inflammations^60^. Further, the limonene was used as a wetting, dispersion, resins, and dissolving agent. Small quantities of 3-pinanylamine were detected in all specimens ranging between 0.21–0.90%. This branched monoterpene hydrocarbon was used to manufacture insecticides and solvents^61^.

#### Hydrocarbons/alcohols/esters/amines/fatty acids/sterols

The minor elements of the FSEO were hydrocarbons (0.29–0.63%). Alcohols were present in amounts ranging from 0.08% in FM + LP to 0.83% in FM. Linalool, which was highly abundant in PM (0.72%); followed by PM + LL compared to other treatments (Table 1), is responsible for the aroma of clementine peel oil which and can be utilized as a flavoring agent owing to its outstanding floral balmy odor^62^.

Esters were abundant in PM (0.81%) compared to other fennel specimens, and amines were present in traces in all fennel treatments (0.01–0.07%) except in PM and PM + LL, which reached to 0.26 and 0.19% respectively. Fatty acids were found in all fennel treatments ranging from 0.47 to 1.25% and other minor constituents of the FSEO were sterols ranging 0.06–0.16% in all fennel treatments, these components contributed to the overall aroma of fennel.

In conclusion, EOs obtained by hydro-distillation were rich in *(E)*-anethole (78.9–91.4%), fenchone (3.35–10.1%), limonene (2.94–8.62%) and estragole (0.50–4.29%) when fennel plants were treated with BOF (Fig. S3). These compounds are responsible for the most intense odor in fennel seed oil. The last compounds were better obtained by treating fennel with the combinations of PM + LP + LL, FM + LP + LL, PM + LL and FM + LL. For this reason, fennel treated with organic and biofertilizers can be used to obtain volatile plant oil at an analytical scale and to obtain FSEO industrially in replacement of traditional techniques based on treated fennel with chemical fertilizers.

### Biological potential of FSEO

#### Antioxidant activity—DPPH assay

An attractive area of nutritional and pharmacological study is analyzing the antioxidant properties of significant oils as lipophilic secondary metabolites. Natural compounds derived from plants are increasingly replacing synthetic food additives because they are safe, efficient, and well-liked by consumers^63^. Fennel, as an edible and medicinal plant, generally denotes importance in the neutralization of reactive oxygen species due to the existence of various secondary metabolites in the fennel oil. This would significantly contribute to their biochemical activities to prevent damage to lipid, DNA, and protein which is thought to be the principal cause of cell aging, oxidative stress-related infections (neurodegenerative and cardiovascular diseases) and cancer^64^. FSEO exhibits high antioxidant activity related to the positive control, l-ascorbic acid (Table 2). The IC_50_, which is defined as the substance concentration which causes a loss of 50% of the DPPH activity (color)^65^, was the criterion employed to measure the DPPH FRSA.

**Table 2:** Antioxidant potential of FSEO that is determined through DPPH assay.

Samples	IC_50_ (μg mL^−1^)
Control	26.54 ± 0.01^b^
FM	25.90 ± 0.01^c^
FM + LP	20.88 ± 0.01^d^
FM + LL	15.12 ± 0.01^f^
FM + LP + LL	09.01 ± 0.01^h^
PM	25.10 ± 0.01^c^
PM + LP	16.12 ± 0.01^e^
PM + LL	12.15 ± 0.01^g^
PM + LP + LL	08.11 ± 0.01^i^
l-ascorbic acid	35.90 ± 0.01^a^

Treatments were: (1) C, control, no seed or soil treatment; (2) FM, soil treatment with farmyard manure; (3) FM + LP, soil treatment with farmyard manure + seed treatment with *Lactobacillus plantarum*; (4) FM + LL, soil treatment with farmyard manure + seed treatment with *Lactococcus lactis*; (5) FM + LP + LL, soil treatment with farmyard manure + seed treatment with *Lactobacillus plantarum* + *Lactococcus lactis*; (6) PM, soil treatment with poultry manure; (7) PM + LP, soil treatment with poultry manure + seed treatment with *Lactobacillus plantarum*; (8) PM + LL, soil treatment with poultry manure + seed treatment with *Lactococcus lactis*; (9) PM + LP + LL, soil treatment with poultry manure + seed treatment with *Lactobacillus plantarum* + *Lactococcus lactis.*

The values expressed as means (n = 3). Based on the Duncan’s multiple range test at *P* ≤ 0.05; the means of rows sharing different small letters (a–i) are significantly different. IC_50_, the half-maximal inhibitory concentration (IC_50_) values (the concentration required for 50% inhibition of viability).

FSEO, fennel seeds essential oil; DPPH, 2,2-diphenyl-1-picrylhydrazyl.

There was a noticeable difference in FSEO FRSA (Table 2). The lower values of IC_50_ (for DPPH), the higher antioxidant activities. The triple combinations of PM + LP + LL and FM + LP + LL had the most significant antioxidant effect, with the lowest IC_50_ values of 8.11 ± 0.01 and 9.01 ± 0.01 μg mL^−1^, respectively, which were about four- and three-fold higher than l-ascorbic acid (IC_50_ = 35.90 ± 0.01 μg mL^−1^; Table 2). Among all nine treatments, control had the lowest DPPH FRSA (IC_50_ = 26.54 ± 0.01), which corresponds to 1.35 times lower than of l-ascorbic acid. These IC_50_ values in fennel plants treated with triple combinations of PM + LP + LL and FM + LP + LL were found to be relatively superior antioxidants to the Egyptian *F. vulgare* treated only with organic fertilizer (compost) whose IC_50_ value was 15.3 mg mL^−1^^66^, Iranian *F. vulgare* var. *vulgare* (IC_50_ = 15.33 mg mL^−1^)^67^, Chinese fennel (IC_50_ = 15.66 mg mL^−1^)^44^, Tajikistan fennel (IC_50_ = 15.6 mg mL^−1^)^64^. Sixteen *F. vulgare* were collected from different regions of Tunisia (IC_50_ ranged from 12 to 38.13 mg mL^−1^)^43^, Pakistani fennel (IC_50_ = 32.32 mg mL^−1^)^41^ and Spanish organic fennel (IC_50_ = 45.89 mg mL^−1^)^1^.

Furthermore, the antioxidant potential of *F. vulgare* treated with the triple combinations was stronger than that of Egyptian *F. vulgare* untreated (IC_50_ = 141.82 mg mL^−1^)^44^. This variation in IC_50_ values was probably due to the treatments of organic and biofertilizers together. This led to differences in the content of the main component (*E*)-anethole which recorded a significantly higher concentration in triple combinations of PM + LP + LL or FM + LP + LL (Table 1). This led to differences in the content of the main component (*E*)-anethole which recorded a significantly higher concentration in triple combinations (Table 1). However, lower values have been reported for untreated Egyptian (46.26%)^66^, Chinese (54.26%)^44^, and Tajikistan (36.8%)^64^.

Except for *(E)*-anethole and estragole, all nine FSEO have comparable concentrations of all other significant components, which shows that the antioxidant activity was mainly related to *(E)*-anethole concentration. One of the main distinctions between the chemical composition of *(E)*-anethole and estragole is the double bond of the propenyl side chain in *(E)*-anethole that is conjugated with the aromatic ring. In contrast, it is nonconjugated in estragole. Contrary to estragole, which can only produce homobenzylic radical cation (Fig. S4), *(E)*-anethole readily forms a conjugated radical cation, which can be delocalized with the aromatic ring and is more stable by the methoxy group through the 1,4 interactions. This variation among *(E)*-anethole and estragole was also seen in their photochemical and free radical dimerization, where anethole dimerized but not estragole by forming the intermediate radical cation^68,69^. This observation may explain the variations in antioxidant activity between the studied FSEO.

As a result, the current work provides for the first time the IC_50_ for FSEO treated with organic and biofertilizers as an evaluation of their antioxidant activity (Table 2). The present study emphasized that FSEO demonstrates the ability as the primary antioxidant interacting with free radicals and inhibiting or scavenging free radicals from the human body; thus, preventing their damage. In addition, it may be concluded that estragole is an excellent alkylation agent while *(E)*-anethole is a better radical scavenger, which may explain that estragole is suspected to be carcinogenin because it can easily alkylate DNA molecules and establish covalent bonds with DNA bases^70^.

#### Antimicrobial effect of FSEO against pathogenic bacteria and fungi

##### Antibacterial potential

The antibacterial activities of FSEO were assessed against four food-borne pathogenic bacteria (*S. aureus, B. subtilis, E. coli*, *and P. aeruginosa*). Based on the inhibition zone diameters obtained, our results were divided into three categories according to^71^: Resistant (< 7 mm), intermediate (> 12 mm) and senstive (> 18 mm).

All FSEO samples, from the current study, exhibited significant antibacterial activities against all the tested strains except for *P. aeruginosa*. FSEO from PM + LP + LL and FM + LP + LL were the most efficient against all tested strains which gave a larger inhibition zone than gentamycin by (23.5%, 25.0%, 6.6% and 16.6%) and (13.3%, 25.0%, 0% and 0%) for *S. aureus, B. subtilis, E. coli* and *P. aeruginosa*, respectively, when 10 µL disk^−1^ was provided (Table 3).

**Table 3:** Antibacterial effect of different FSEO treatments evaluated by disk diffusion assay.

Treatment	Concentration (µL disk^−1^)	Diameter of inhibition zone (mm) of bacteria			
		Gram positive		Gram negative	
		*S. aureus*	*B. subtilis*	*E. coli*	*P. aeruginosa*
Control	10	10 ± 0.76^k^	10 ± 0.30^k^	0.0 ± 0.0^i^	NA
	20	10 ± 0.50^k^	10 ± 0.50^k^	10 ± 0.10^h^	NA
FM	10	11 ± 0.25^j^	10 ± 0.40^k^	12 ± 0.82^f^	NA
	20	11 ± 0.76^i^	10 ± 0.20^k^	12 ± 0.95^f^	NA
FM + LP	10	12 ± 0.50^h^	12 ± 0.30^i^	12 ± 0.40^f^	NA
	20	15 ± 0.40^e^	17 ± 0.45^e^	14 ± 0.25^d^	NA
FM + LL	10	12 ± 0.38^h^	14 ± 0.15^h^	12 ± 0.33^g^	NA
	20	15 ± 0.64^e^	19 ± 0.25^d^	14 ± 0.15^d^	NA
FM + LP + LL	10	15 ± 0.80^e^	16 ± 0.35^f^	14 ± 0.30^d^	10 ± 0.10^c^
	20	20 ± 0.75^b^	22 ± 0.16^b^	16 ± 0.20^b^	12 ± 0.10^b^
PM	10	13 ± 0.50^g^	12 ± 0.90^i^	12 ± 0.62^f^	NA
	20	15 ± 0.40^e^	17 ± 0.55^e^	13 ± 0.14^e^	NA
PM + LP	10	14 ± 0.45^f^	12 ± 0.10^j^	12 ± 0.02^g^	NA
	20	16 ± 0.10^d^	15 ± 0.15^g^	14 ± 0.10^d^	NA
PM + LL	10	12 ± 0.30^h^	15 ± 0.22^g^	12 ± 0.05^g^	NA
	20	17 ± 0.20^c^	20 ± 0.50^c^	16 ± 0.20^b^	NA
PM + LP + LL	10	17 ± 0.40^c^	16 ± 0.22^f^	15 ± 0.15^c^	12 ± 0.10^b^
	20	22 ± 0.10^a^	23 ± 0.12^a^	18 ± 0.04^a^	13 ± 0.10^a^
Gentamycin	10	13 ± 0.12^g^	12 ± 0.24^j^	14 ± 0.03^d^	10 ± 0.05^c^

Treatments were: (1) C, control, no seed or soil treatment; (2) FM, soil treatment with farmyard manure; (3) FM + LP, soil treatment with farmyard manure + seed treatment with *Lactobacillus plantarum*; (4) FM + LL, soil treatment with farmyard manure + seed treatment with *Lactococcus lactis*; (5) FM + LP + LL, soil treatment with farmyard manure + seed treatment with *Lactobacillus plantarum* + *Lactococcus lactis*; (6) PM, soil treatment with poultry manure; (7) PM + LP, soil treatment with poultry manure + seed treatment with *Lactobacillus plantarum*; (8) PM + LL, soil treatment with poultry manure + seed treatment with *Lactococcus lactis*; (9) PM + LP + LL, soil treatment with poultry manure + seed treatment with *Lactobacillus plantarum* + *Lactococcus lactis*.The values are the means (n = 3). Values with the same letter within a column for treatment are not significantly (*P* > 0.05) different according to Duncan’s multiple range test.

FSEO, fennel seeds essential oil; NA, no activity. *S. aureus*, *Staphylococcus aureus*; *B. subtilis*, *Bacillus subtilis*; *E. coli*, *Escherichia coli*; and *P. aeruginosa*, *Pseudomonas aeruginosa.*

Our findings also showed that *S. aureus* and *B. subtilis* were the most sensitive bacteria tested, revealing the largest inhibition zones, while the smallest inhibition zone was for *E. coli* (Table 3). None of the studied FSEO effectively inhibited *P. aeruginosa* except that of PM + LP + LL and FM + LP + LL. Our results are in alignment with another study^72^, suggesting that FSEO has considerable antibacterial activity, particularly towards Gram positive bacteria compared to Gram negative isolates. According to^73^, FSEO inhibits various *Bacillus* species and had less sensitivity to Gram negative bacteria.

It has been reported that these differences between Gram-positive and Gram-negative bacteria are caused by their distinct cell walls^74–76^. Such variations alter plasma coagulation, cause DNA destruction, modify enzymatic processes or increase plasma membrane permeability, which may result in greater leakage of fluid material from bacterial cells^77^ and decrease microbial respiration^78^.

In conclusion, the treated FSEO with any of the triple combinations was highly effective against Gram positive and negative bacteria, and may be employed as a natural antibacterial agent for treatments of several infectious disorders initiated by these pathogenic bacteria.

##### Antifungal potential

**Figure 3:**
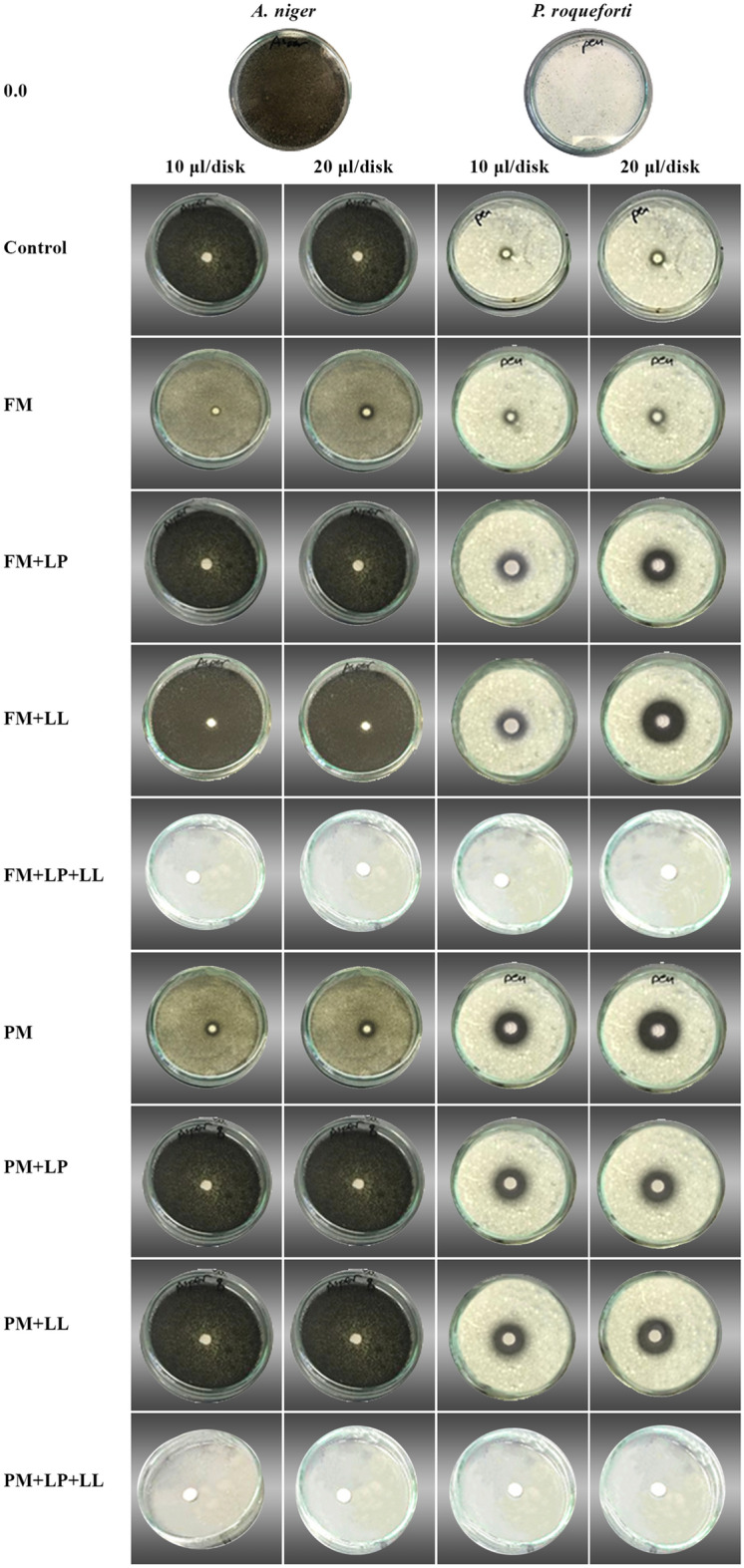
Effect of FSEO concentration on the mycelial growth of *Aspergillus niger* and *Penicillium roqueforti*. Treatments were: (1) C, control, no seed or soil treatment; (2) FM, soil treatment with farmyard manure; (3) FM + LP, soil treatment with farmyard manure + seed treatment with *Lactobacillus plantarum*; (4) FM + LL, soil treatment with farmyard manure + seed treatment with *Lactococcus lactis*; (5) FM + LP + LL, soil treatment with farmyard manure + seed treatment with *Lactobacillus plantarum* + *Lactococcus lactis*; (6) PM, soil treatment with poultry manure; (7) PM + LP, soil treatment with poultry manure + seed treatment with *Lactobacillus plantarum*; (8) PM + LL, soil treatment with poultry manure + seed treatment with *Lactococcus lactis*; (9) PM + LP + LL, soil treatment with poultry manure + seed treatment with *Lactobacillus plantarum* + *Lactococcus lactis*. FSEO, fennel seed essential oil.

FSEO components were more effective and showed more fungicidal potential than clotrimazole (Fig. 3; Table 4). FSEO produced a complete zone of inhibition relative to the standard drug for *A. niger*. It also has the same higher activity against *P. roqueforti*, forming a zone of inhibition larger than the standard drug by 100%.

**Table 4:** Effect of FSEO on the mycelial growth of fungal isolates.

Treatment	Concentration (µL disk^−1^)	Zone of inhibition (mm)	
		*Aspergillus niger*	*Penicillium roqueforti*
	0.0	NA	NA
Control	10	NA	10 ± 0.03^h^
	20	NA	10 ± 0.01^h^
FM	10	12 ± 0.10^c^	10 ± 0.05^h^
	20	15 ± 0.01^b^	15 ± 0.03^f^
FM + LP	10	NA	14 ± 0.01^g^
	20	NA	17 ± 0.04^e^
FM + LL	10	NA	15 ± 0.02^f^
	20	NA	22 ± 0.10^b^
FM + LP + LL	10	CI ± 0.00^a^	CI ± 0.00^a^
	20	CI ± 0.00^a^	CI ± 0.00^a^
PM	10	12 ± 0.12^c^	15 ± 0.16^f^
	20	15 ± 0.10^b^	20 ± 0.12^c^
PM + LP	10	NA	15 ± 0.02^f^
	20	NA	15 ± 0.01^f^
PM + LL	10	NA	15 ± 0.04^f^
	20	NA	18 ± 0.06^d^
PM + LP + LL	10	CI ± 0.00^a^	CI ± 0.00^a^
	20	CI ± 0.00^a^	CI ± 0.00^a^
Clotrimazole	10	12 ± 0.01^c^	10 ± 0.05^h^

Treatments were: (1) C, control, no seed or soil treatment; (2) FM, soil treatment with farmyard manure; (3) FM + LP, soil treatment with farmyard manure + seed treatment with *Lactobacillus plantarum*; (4) FM + LL, soil treatment with farmyard manure + seed treatment with *Lactococcus lactis*; (5) FM + LP + LL, soil treatment with farmyard manure + seed treatment with *Lactobacillus plantarum* + *Lactococcus lactis*; (6) PM, soil treatment with poultry manure; (7) PM + LP, soil treatment with poultry manure + seed treatment with *Lactobacillus plantarum*; (8) PM + LL, soil treatment with poultry manure + seed treatment with *Lactococcus lactis*; (9) PM + LP + LL, soil treatment with poultry manure + seed treatment with *Lactobacillus plantarum* + *Lactococcus lactis*.Values with the same letter within a column for each treatment are not significantly (*P* > 0.05) different according to Duncan’s multiple range test.

FSEO, fennel seeds essential oil; CI, complete inhibition; NA, no activity.

**Figure 4:**
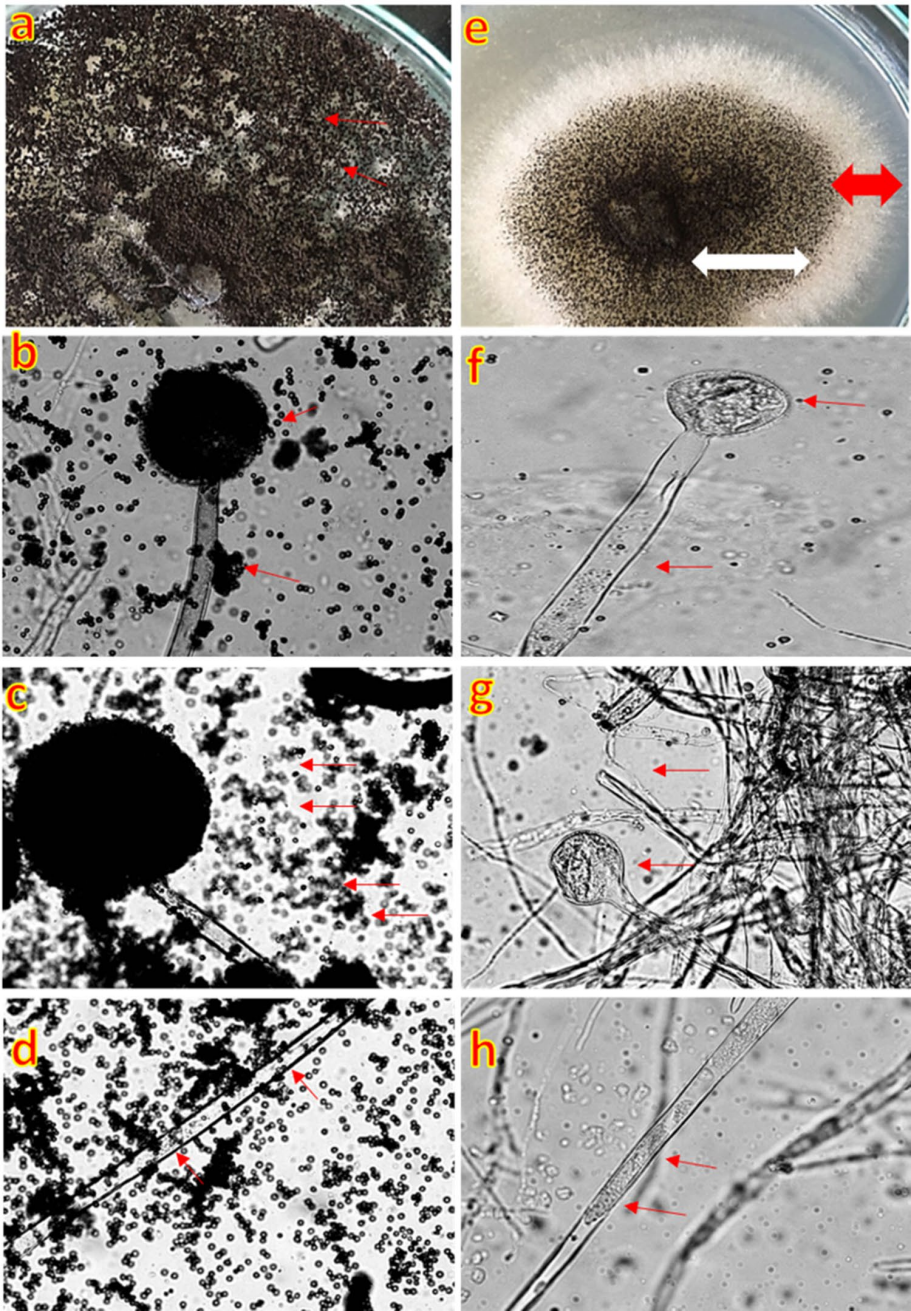
Effect of FSEO on the mould, *Aspergillus niger, *under the light microscope. *A. niger* growing in plates to determine the impact (**a**–**d**) without (control) or (**e**–**h**) with FSEO on the (**a**,**e**) morphological characteristics of hyphae; (**b**,**f**) sporangiophores; (**c**,**g**) number of spores; and (**d**,**h**) thickness and elongation of hyphae. Note that FSEO showed (**e**) inhibition of fungal growth (white arrow) and sporulation (red arrow); (**f**) deformed sporangiophore; (**g**) absence of spores, and (**h**) cytoplasmic coagulation in hyphae. Light micrographs of *A. niger* hyphae were exposed to FSEO at ×40. FSEO, fennel seed essential oil.

The effect of FSEO has been tested against *A. niger* mycelial growth. FSEO reduced mycelial growth of *A. niger* because there was no fungal sporulation on the 5th day of the FSEO-treated sample compared to the control without FSEO. Light microscopic examinations supported these findings. Microscopic observation of *A. niger* hyphae exposed to FSEO showed hyphal morphological changes compared to normal morphology in control hyphae (Fig. 4). Compared with thick, elongated and normal mycelial growth in controls (Fig. 4a–d), hyphae appeared thinner with cytoplasmic coagulation and looked empty as if the hyphal cells drained up from cytoplasm and organelles (Fig. 4f–h). We did not observe conidiospores under the microscope (Fig. 4f,g). The mechanism of action of volatile oil^39,79^ can be attributed to the hyphal morphological changes which may be a result of the lipophilic character of EOs that gives them the ability to easily penetrate the fungal mycelia causing cell integrity loss and deformation of fungal mycelia. Secondly, their significant components' effect might increase the plasma membrane's permeability, resulting in hyphal function disorders and deformation.

## Conclusion

For the first time, the current study presents variability in the FSEO concentration and chemical composition of Egyptian *F. vulgare* seeds grown in saline calcareous-soil treated with OM and biofertilization, as well as their combinations. High oil yield and higher content of the medicinal and culinary compounds *(E)*-anethole (78.9–91.4%), fenchone (3.35–10.19%), limonene (2.94–8.52%), and estragole (0.50–4.29%) were observed in *F. vulgare* fertilized with any of the triple combinations. According to the DPPH assay, the antioxidant activity of FSEO treated with PM + LP + LL and FM + LP + LL was four- and three-times higher than that of l-ascorbic acid, respectively. Compared to other treatments, FSEO treated with triple combinations showed relatively superior antimirobial activity against pathogenic bacteria and fungi. To achieve the highest yields, chemical constituents, and biological activities of FSEO, it is recommended that researchers must supply fennel plants with a mixture of OM with biofertilizers of *L. plantarum* subsp*. plantarum* and *L. lactis* subsp*. lactis*. As a sustainable approach, fennel breeders can benefit from this research by combining OM and biofertilizers without addition of chemical fertilizers and treatment of soils; thus, achieving remarkable progress in fennel genotypes with high salinity tolerance, strong aroma, and excellent biological, therapeutic, and pharmaceutical effects. The findings of this research could be of use to consumers as a risk-free alternative to non-organic fennel in the production of food additives and commercial formulations that use FSEO as a source of natural antioxidants and antimicrobials.

### Supplementary Information

Supplementary Information.

## Data Availability

The data presented in this study are available upon request from the corresponding author.
