# Supplementary material for: Bio-organic fertilizers promote yield, chemical composition, and antioxidant and antimicrobial activities of essential oil in fennel (Foeniculum vulgare) seeds
Source: Sci Rep. 2023 Aug 25;13:13935. doi: 10.1038/s41598-023-40579-7 (PMC10457370; doi:10.1038/s41598-023-40579-7)
Supplement: Supplementary file 1 — Supplementary Information. [file 41598_2023_40579_MOESM1_ESM.pdf]

Supplementary Data

Bio-organic Fertilizers Promote Yield, Chemical Composition, and Antioxidant and Antimicrobial Activities of Essential Oil in Fennel (*Foeniculum vulgare*) Seeds

Ahmed S. Abdelbaky, Abir M.H.A. Mohamed, Taia A. Abd El-Mageed, Mostafa M. Rady, Fatma Alshehri, Mohamed T. El-Saadony, Synan F. AbuQamar*, Khaled A. El-Tarabily*, Omar A. A. Al-Elwany

*** Correspondence:**

Prof. Synan AbuQamar: sabuqamar@uaeu.ac.ae

Prof. Khaled El-Tarabily: ktarabily@uaeu.ac.ae

Table S1. Physicochemical parameters of the experimental soil used in the current study.

Soil characteristics	Values	Soil characteristics	Values
**Chemical properties**		**Available nutrients**	
pH	7.64	N	0.16%
ECe	6.92 dS m^{-1}	P	83.51 mg kg^{-1} dry soil
OM	0.89%	K	170.6 mg kg^{-1} dry soil
$CaCO_3$	13.8%		
		**Physical properties**	
**Exchangeable cations**		Coarse sand	50.44%
Ca^{+2}	9.67 meq L^{-1}	Fine sand	24.22%
Mg^{+2}	7.26 meq L^{-1}	Clay	13.19%
Na^{+}	26.3 meq L^{-1}	Silt	12.15%
K^{+}	3.14 meq L^{-1}	Soil type	Sand loamy

ECe, electrical conductivity; OM, organic matter; N, nitrogen; P, phosphorus; K^{+}, potassium; Ca^{+2} calcium; Mg^{+2}, magnesium; and Na^{+}, sodium

Table S2. Chemical properties of farmyard manure (FM) and poultry manure (PM).

**Properties**	FM	PM
pH	7.56	7.42
ECe (dS m^{-1})	3.98	4.50
OM (%)	39.5	45.6
N (%)	1.16	1.36
P (%)	0.57	0.49
K (%)	1.89	1.57

ECe, electrical conductivity; OM, organic matter; N, nitrogen; P, phosphorus; and K, potassium.

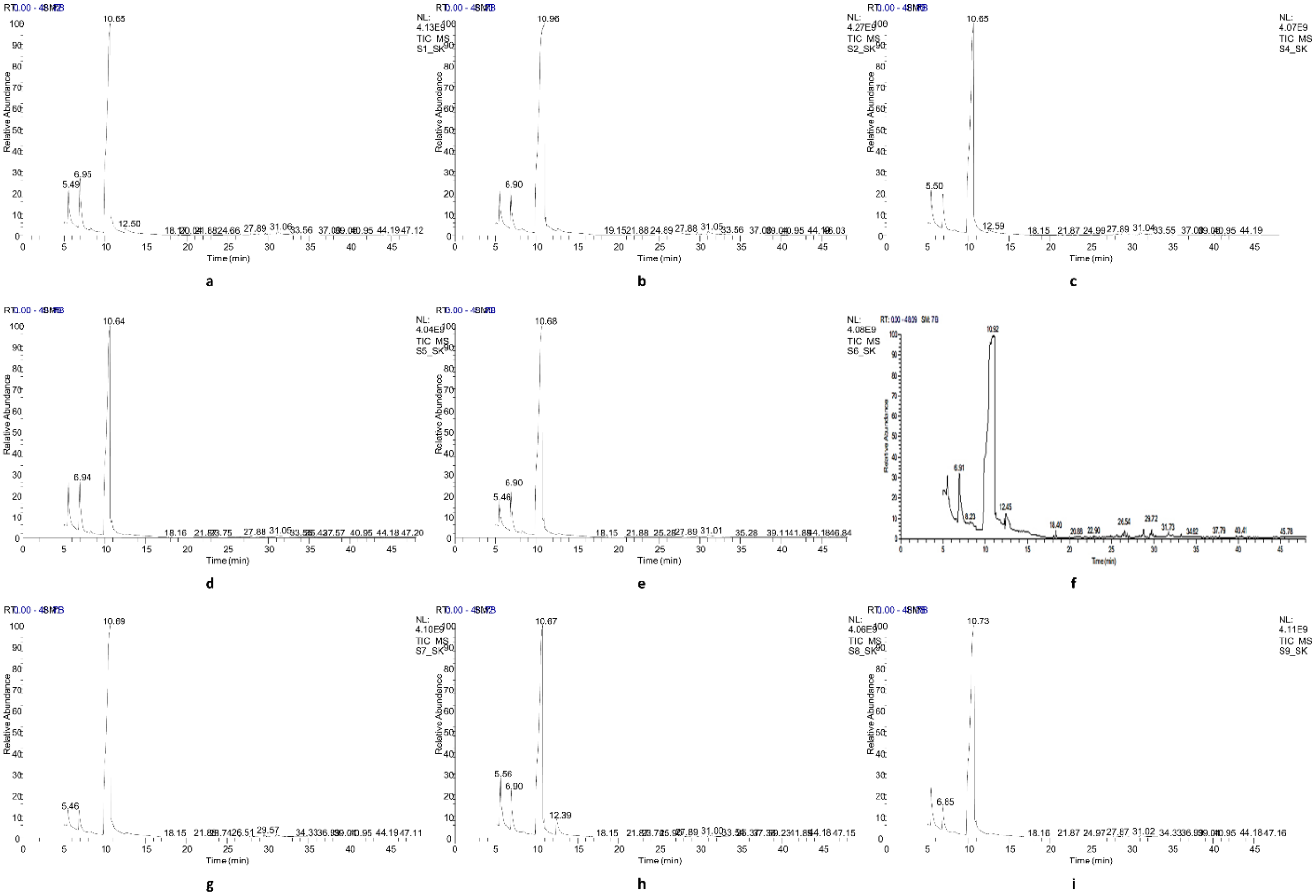

Figure S1. Gas chromatography–mass spectrometry analysis of the composition of fennel seeds essential oil (FSEO) from (a) Control, (b) FM, (c) FM+LP, (d) FM+LL, (e) FM+LP+LL, (f) PM, (g) PM+LP, (h) PM+LL, and (i) PM+LP+LL. FM, farmyard manure; PM, poultry manure; LP, *Lactobacillus plantarum*; LL, *Lactococcus lactis*.

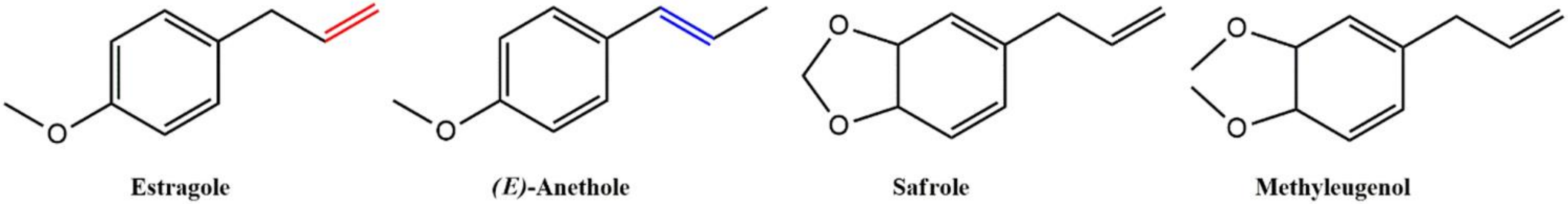

Figure S2. Chemical structures of alkenylbenzenes estragole, *(E)*-anethole, safrole and methyleugenol.

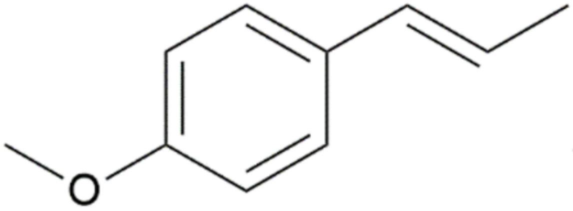

***(E)*-anethole**

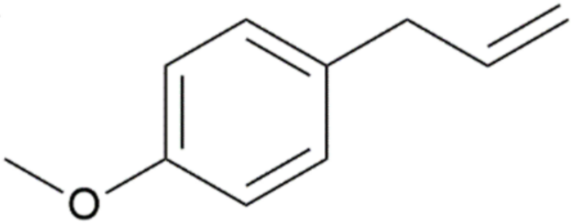

Estragole

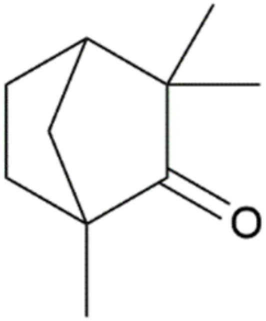

Fenchone

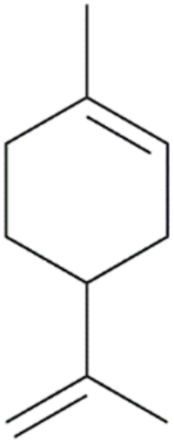

Limonene

Figure S3. Structures of the main compounds of fennel seed essential oil (FSEO).

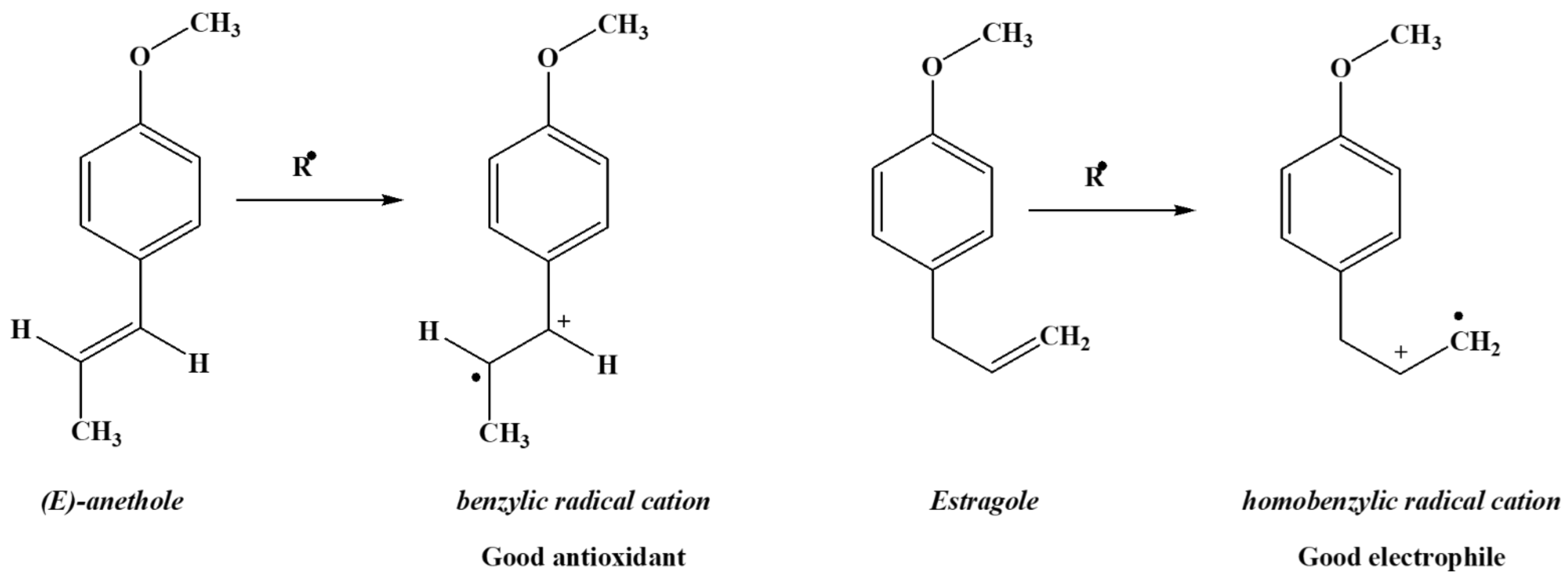

Figure S4. Radical intermediates derived from *(E)*-anethole and estragole.
